# Electron Cryo‐Microscopy of TPPS_4_⋅2HCl Tubes Reveals a Helical Organisation Explaining the Origin of their Chirality†

**DOI:** 10.1002/cphc.201300606

**Published:** 2013-07-31

**Authors:** Judith M. Short, John A. Berriman, Christian Kübel, Zoubir El‐Hachemi, Jean‐Valère Naubron, Teodor Silviu Balaban

**Affiliations:** ^1^ Division of Structural Studies, MRC Laboratory of Molecular Biology, Francis Crick Avenue, Cambridge, CB1 0QH (UK); ^2^ Karlsruhe Institute of Technology (KIT), Institute of Nanotechnology (INT), Hermann‐von‐Helmholtz‐Platz 1, 76344 Eggenstein‐Leopoldshafen (Germany); ^3^ Department of Organic Chemistry University of Barcelona, Catalonia (Spain); ^4^ Aix Marseille Université, Spectropole, CNRS FR 1739, Avenue Escadrille Normandie Niemen, Marseille (France); ^5^ Aix Marseille Université, Institut des Sciences Moléculaires de Marseille (iSm2), CNRS UMR 7313, Chirosciences, Avenue Escadrille Normandie Niemen, Case A62, 13397 Marseille CEDEX 20 (France)

**Keywords:** chirality, electron cryo‐microscopy, helical reconstruction, J‐aggregates/H‐aggregates, porphyrinoids, self‐assembly

## Abstract

**A widely studied** achiral porphyrin, which is highly soluble in aqueous solutions (TPPS_4_), is shown to self‐assemble into helical nanotubes. These were imaged by electron cryo‐microscopy and a state‐of‐the‐art image analysis allows building a map at ∼5 Å resolution, one of the highest obtained so far for molecular materials. The authors were able to trace the apparent symmetry breaking to existing nuclei in the “as received samples”, while carefully purified samples show that both handnesses occur in equal amounts.

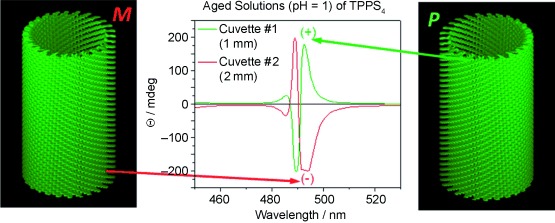

The conversion of solar energy into heat, electricity or fuels to meet future changes in the world’s resources is currently an area of intense study and research.^1^ Bionics offers engineers new approaches to design developments and applications; nature itself has evolved systems of efficient and highly diverse light‐harvesting assemblies of chromophores which are adapted to the illumination conditions of their local environment.^2^ Photosynthesis is an inspiring blueprint which could help solve the energy problem and curb carbon dioxide emissions related to burning fossil fuels.^3^

Light‐harvesting J‐aggregates are named after Jelley^4^ and were independently discovered and accurately described by Schiebe.^5^ These supramolecular assemblies of chromophores have different optical properties from their constituent monomers. The offset head‐to‐tail packing is responsible for a red‐shifted absorption at longer wavelengths. The related H‐aggregates have the transition dipole moments oriented parallel and face‐to‐face which leads to a blue‐shifted absorption. Such aggregates have been intensively studied both from a fundamental point of view and for applications related to light‐harvesting.^6^

A most controversial system is that of acidic aqueous solutions of achiral *meso*‐tetrakis‐(4‐sulfonanophenyl)porphyrin (TPPS_4_). Despite the use of a wide variety of physical techniques, no definitive structure has yet been determined to elucidate the supramolecular interactions of the intensely green J‐aggregates which form in solutions at pH<4 of TPPS_4_^7^ or of closely related compounds, such as *meso*‐5‐phenyl‐10,15,20‐tris‐(4‐sulfonatophenyl)porphyrin (TPPS_3_),^8^ or the sulfonatothienyl analogues.^9^ Identifying the orientation and packing between molecules is vital for understanding the driving force behind the aggregate formation and also for controlling and fine‐tuning the photophysical properties. The ability to discriminate between theoretical models which try to explain the shifts found in absorption spectra is crucial given the current wide speculation with more than 1200 references to TPPS_4_ as of April 2013. Most recently, Ribó and co‐workers have carried out an extensive X‐ray study of aligned samples together with electron diffraction data from dried samples and have interpreted the diffraction intensities as stemming from extended two‐dimensional (2D) sheets of TPPS_4_ molecules.^10^

Another recent model was proposed by Russo and coworkers based on a small angle X‐ray scattering (SAXS) study combined with analytical centrifugation,7a reinforcing the pioneering SAXS study of Tabak and coworkers7b In both these studies, due to the inherently low resolution and uncertainties of this technique, it was impossible to detect any trace of helicity in the tubular structures. Proving supramolecular helicity is important as it provides an explanation for the chirality evident in the electronic circular dichroism (ECD) spectra. This chirality stands as an enigmatic example of symmetry breaking by an achiral, apparently C_4*V*_ symmetric, TPPS_4_ monomer. Self‐assembly can produce preparations with mirror‐imaged exciton couplets in the ECD spectra around the wavelength region where the aggregate absorbs light strongly (J‐ and H‐bands). This bias of chirality and symmetry breaking has been demonstrated as depending on “Butterfly Effect” influences such as the rotational direction of a rotary evaporator, a stirring vortex mixer and strong magnetic fields for forming enantiomorphic superstructures.7m, 8, 11 We stress here that this chirality induction operates at a strictly supramolecular level and is inoperative at the small, covalent molecular level, for instance in vortex biasing enantioselective reactions from achiral reagents or catalysts. Consequently, over a wide range of interests in soft matter physics and chemistry, the structure of TPPS_x_ aggregates is a highly relevant question.

Electron microscopy (EM) has previously been used to characterise TPPS_4_ aggregates coated by silica and dried in a vacuum which were described as nanotapes.^12^ Influenced strongly by X‐ray crystallography, structural biology has driven EM to accommodate the important contribution to supramolecular integrity made by water.^13^ Cryo‐EM maintains the aqueous milieu in a vitreous state where complex structures are hydrated, embedded and thereby physically supported.^14^ It has previously been applied to J‐aggregates by Knoester et al. who reported large numbers of long thin‐walled nanotubes in acidic TPPS_4_ solutions. Their absorption spectra were calculated and tested against possible models but no image analysis was described.^15^ Not only did this indicate that the tubes collapsed to a tape when dried but also, being composed of organic molecules, they were delicate and sensitive to radiation damage. We decided to apply cryo‐EM diffraction methods to determine the level of structural order in the tubes, then measure the sensitivity to irradiation. The optimal settings of magnification, beam intensity and exposure time were then used for low dose imaging of individual tubes. The low dose technique is a compromise between radiation damage and resolution which produces images with a low signal‐to‐noise ratio. It is then necessary to select tubes by the quality of their power spectra rather than attempting to inspect the crystalline lattice directly. It is important to average together as many long, straight tubes as possible; the larger the number of undistorted subunits and orientations, the higher the resolution. The well‐established method of helical Fourier‐Bessel analysis^16^ has the advantage of being able to exploit both phase and amplitude information. We used the MRC helical programs^17^ to calculate a three‐dimensional (3D) model of the TPPS_4_ tubes to ∼5 Å resolution which was sufficient to determine the packing of the porphyrins within the wall of the tube.

TPPS_4_ isolated as the dihydrochloride salt, dissolved in water at neutral pH, shows a monomeric absorption spectrum of a diprotonated species having an intense green colour due to the Q bands peaking at 646 nm (Supporting Information, Figure S1). The much more intense Soret band is at 433 nm. These bands are typical for a free base porphyrin (TPPS_4_⋅2 HCl) with the two pyrrolic and and two pyrrolenic nitrogens protonated as indicated in Scheme 1. Upon lowering the pH, both a sharp intense J‐aggregate band appears at 490 nm, and a broader, less intense H‐band is visible as a shoulder, blue shifted from the monomeric Soret band of the diprotonated species at 433 nm (Figure 1 A). In the Q bands region, the J‐aggregates have an absorption maximum at 706 nm. Upon dilution with pH 1 aqueous HCl, the monomer maxima gradually increase while the aggregate peaks decrease abruptly proving the existence of an equilibrium (Figure S1).^15^ Also shown in Figure 1 C are two mirror imaged ECD spectra. While these are silent for the monomeric bands, both J‐ and H‐aggregates give intense Cotton effects after a ripening period of about one day. Any structural model should also explain the optical properties of the TPPS_4_ aggregates, including the observed equilibria and growth mechanism during the ripening phase.

**Scheme 1:**
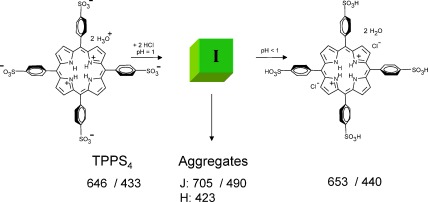
Protonation of the inner pyrrolenic nitrogen atoms and of two sulphonic groups at low pH (100 mm HCl) leads to an intermediate **I** which self‐assembles forming aggregates in water. At pH<1 all four sulphonic groups are protonated.7k Numbers indicate absorption maxima in nm.

**Figure 1:**
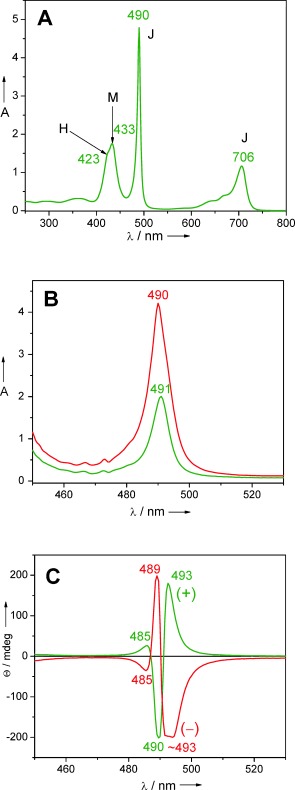
A) Aggregation behaviour after injection of 300 μL of a solution 5.3 mg TPPS_4_⋅2 HCl dissolved in 1.0 mL deionized water (pH 7) into 10 mL aqueous HCl at pH 1. **M** denotes monomer maxima, **J** and **H** denote aggregate maxima. B,C) Absorption and ECD spectra, respectively, of samples prepared from similar aggregation experiments, ripened for 24 h. The rarely encountered negative exciton couplet (red trace) has the longest Cotton effect slightly truncated due to saturation of the detector. The different cuvette pathlengths (red trace—2 mm; green trace—1 mm) were chosen to obtain similarly intense Cotton effects and were not responsible for the mirror imaged traces.

One of the present authors has studied the aggregation behaviour of bacteriochlorophyll *c* (BChl *c*) and its synthetic mimics in nonpolar solvents.^2^ In this case a concentrated BChl *c* precursor solution in a moderately polar solvent, such as dry dichloromethane, is injected into a much larger volume (at least 50 times) of a nonpolar solvent like *n‐*hexane, cyclohexane or *n*‐heptane. This abrupt change in solvent polarity is followed by a nucleation phase after which large aggregates are formed by self‐assembly via an autocatalytic process.^18^ Kinetics and thermodynamics must be carefully controlled for reproducing size distributions and optical properties. Alternatively, one can induce aggregation of these chlorosomal type pigments by injecting a concentrated (mm) solution in tetrahydrofuran into water in the presence of surfactants.^19^ The use of aqueous solutions is environmentally benign should large scale applications be envisaged and TPPS_4_ is an ideal candidate for light‐harvesting. We used the same injection protocol by passing a millimolar concentrated solution of TPPS_4_⋅2 HCl dissolved in pH 7.0 distilled water into a much larger volume of aqueous HCl at pH 1. This was achieved by injecting slowly, preferably through a capillary and by handling all aqueous solutions in plastic vessels.^20^ In this way we could reproducibly obtain long hollow tubes of TPPS_4_ dihydrochloride with a narrow diameter distribution, which could be successfully imaged by cryo‐EM as shown in Figure 2 A. More recently, other groups have also applied this injection method for inducing self‐assembly to chromophores^21^ and using other analytical techniques they deduced the supramolecular architectures. In such soft matter self‐assemblies, obtaining single crystals for X‐ray diffraction is a daunting task. Although with other self‐assembling porphyrins we could grow a plethora of single crystals,^22^ with aqueous TPPS_4_ samples we failed with systematic trials for more than five years. The aggregation behaviour of TPPS_4_ in aqueous solutions as a function of pH and ionic strength has been carefully and repeatedly studied by varied spectroscopic techniques.7m,n, 23 By our injection method derived from the self‐assembly of BChls and their mimics, we obtain visually homogeneous solutions which show prominent J (at 490 nm) and H (at 423 nm) aggregate maxima and only minor residual monomer peaks (at 433 nm) as shown in Figure 1 and S1.

**Figure 2:**
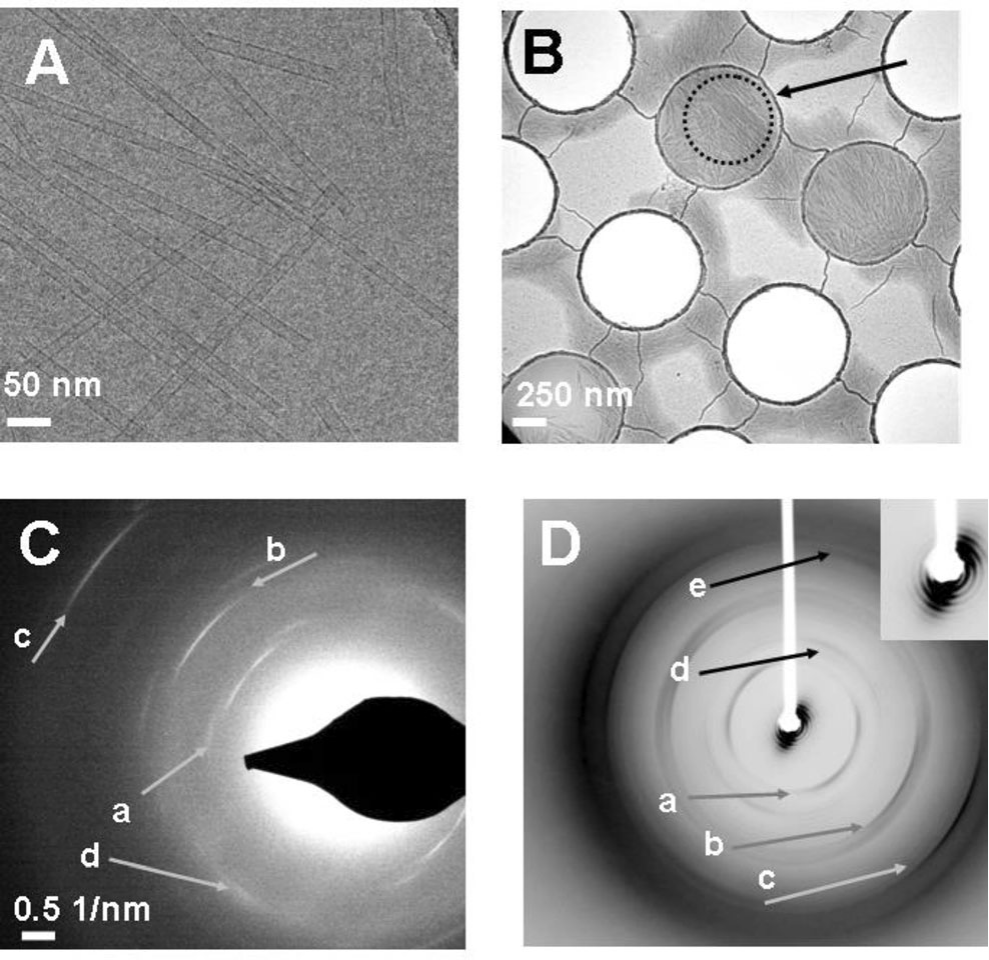
A) Cryo‐EM image of a collection of TPPS4 tubes prepared by the injection method of a concentrated aqueous solution (pH 7 water) into a much larger volume of pH 1.0 aqueous HCl solution. B) TPPS_4_ tubes were concentrated by centrifugation and pipetted on the Quantifoil holey carbon grid for freezing. In this low magnification image some of the 1 μm holes are empty, while bundles of tubes are visible in the ice across the others. One with best alignment was chosen for diffraction and the area circled and arrowed was selected. C) Electron diffraction pattern taken of the selected area showing arcs characteristic of helical organisation with layer lines arrowed at **a**: 4.8, **b**: 3.5, and **c**: 2.1 Å^−1^ spacings while d is an equatorial reflection at 3.7 Å^−1^. D) X‐ray diffraction pattern of a partially aligned preparation of TPPS4 tubes showing (arrows **a**–**c**) 9.4, 5.2 and 3.5 Å^−1^, arrows **d** and **e** show equatorial spacings at 7.4 and 3.7 Å^−1^. Close inspection of the small angle data around the beam stop (inset) shows how the alignment sharpens the rings of diffraction, consistent with the sample being packed tubes of a narrow range of diameters.

In order to confirm that the nanotubes visible by cryo‐EM were indeed aggregated TPPS_4_ molecules, the sample was centrifuged. The preparation separated into an intensely green pellet and a supernatant which had lost all of the optical properties associated with the J‐ or H‐aggregates. Examining the pellet by cryo‐EM revealed closely packed nanotubes which were amenable to electron diffraction of the frozen material in a manner often applied in structural biology.^24^ Following flow‐alignment of the concentrated material, selected regions of the frozen grid (Figure 2 B) yielded electron diffraction patterns characteristic of helical order as shown in Figure 2 C and indicating order at atomic resolution (∼2 Å). Since electron microscopy selects data from extremely small amounts of sample and there is always a danger of possible contamination from airborne dust and fibres, the experiment was scaled‐up 100‐fold to produce sufficient material for X‐ray diffraction. The pelleted TPPS_4_, prepared in the same way as for electron microscopy, was aligned and mounted by flowing through a long thin pipette tip into a 2 mm diameter thin‐walled tube. Although only partially aligned, the pattern shown in Figure 2 D indicates that fundamentally identical results were obtained, confirming the electron microscopy results and also revealing a system of small angle (SAXS) rings around the beam‐stop which is consistent with previous investigations.7a,b

The tubes were numerous, long and reasonably straight but were critically affected by ice thickness: too thin and the tube was flattened and distorted, too thick and the power spectrum was too weak to be useful. The diameters were calculated by the following procedure: each tube was boxed, vertically aligned and its densities summed in the direction of the tube axis. This provided a one‐dimensional trace where the tube boundaries could be measured reasonably accurately (Figure S2). The average diameter calculated from 102 tubes was 159.6 Å with a standard deviation of 7.5 Å. Only a very small percentage of the tubes imaged produced Fourier transforms suitable for indexing and reconstruction. Four tubes were selected with radii varying between 75 Å and 85 Å. Layer lines were observed at around 10.7 Å^−1^, 5.1 Å^−1^ and in one case at 3.6 Å^−1^. The indexing measurements and the repeat distances showed slight variation between the four tubes. Values extracted from the averaged power spectrum shown in Figure S3 were imposed on all eight sides of the four tubes before averaging together the layer line data extracted from the Fourier transform and carrying out the inverse transformation. The tubes were indexed as a 26‐start helix with 72 subunits per turn as shown in Figure 3. In order to confirm the indexing, a larger set of tubes was boxed as segments and classified using the IMAGIC software.^25^ From those class averages which showed a consistent radius, 36 000 overlapping segments of tube images were extracted and subjected to processing by iterative helical single particle analysis (IHRSR)^26^ which itself uses the SPIDER package.^27^ Along with the averaged power spectrum, measurements from many Fourier transforms also indicated that the majority of tubes showed a 26‐start helix. This was fixed for the calculations which converged after 30 iterations to an angular displacement of 5° and a rise of 3.6 Å.

**Figure 3:**
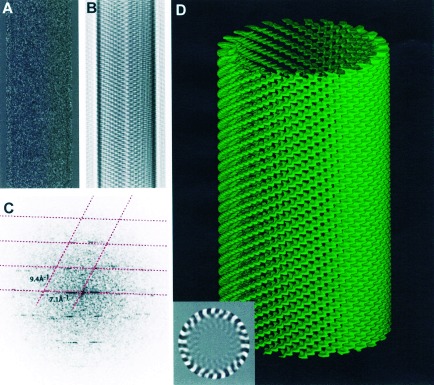
Part of a single tube which was boxed (A), masked and Fourier filtered (B) and Fourier transformed (C). The lattice is shown in (C) as a dashed red line. Three‐dimensional reconstruction (D) computed by helical Fourier‐Bessel analysis of both sides of four tubes averaged together shows the packing of the porphyrin molecules. A single section of the map viewed down the cylinder axis shows the 26 helices (inset). Layer lines at 122 Å^−1^,10.7 Å^−1^ and 5.1 Å^−1^ were included in the calculation. The resolution is ∼5 Å. This is a ***P*** (+) 26‐start helix with 72 subunits in each turn of the helix.

The helical arrangement of the porphyrin molecules translates into an intrinsically chiral arrangement of their transition dipole moments. As a consequence, in the ECD, intense bisignate Cotton Effects are encountered for the J‐aggregate band at 490 nm (Figure 1 C). We note that similar split Cotton effects are encountered in the circular arrangements of BChl *a* in LH2^28^ and LH1,^29^ or in the BChl *c*, *d*, or *e* self‐assemblies typical of chlorosomes.^30^ In this case the BChl chromophores are chiral molecules having several stereogenic carbon atoms and their arrangement generates chiral supramolecular structures which usually have the same chirality induced by the individual molecules.

In the case of TPPS_4_ both chiralities, that is, either ***P*** or ***M*** helices are generated separately from an apparently achiral *C*_4*v*_ symmetric building block.

TPPS_4_ dissolved in pH 7 water, after initial dilution into pH 1 aqueous acid solutions (albeit showing prominent aggregate absorption maxima) displays only weak and random signed ECD signals corresponding to the aggregates. Upon ageing for at least 24 hrs, strong exciton couplets evolve for both the J‐aggregate band at 490 nm and somewhat less intensely for the blue shifted H‐aggregate band. In Marseille, with several batches of commercial TPPS_4_⋅2 HCl samples used “as received”, a strong dominance of a positive exciton couplet, that is a positive Cotton effect at longer wavelength, is encountered before the negative Cotton effect at shorter wavelengths.

This corresponds, according to the exciton chirality method,^31^ to a clockwise (***P***) superposition of the transition dipole moments in neighbouring porphyrins. Out of 80 different aggregation experiments using various TPPS_4_ batches and distilled water sources, only two presented the opposed chirality, that is, a negative exciton couplet (red trace in Figure 1 C). Ribó and co‐workers noted similarly, on a larger number of TPPS_4_ samples in studies spanning a decade, that less than 4 ‰ show the negative exciton couplet.^32^ In a recent study, using achiral ionic liquids and TPPS_4_, symmetry breaking was reported from 50 trials, 36 showing positive exciton couplets and 14 negative ones.^33^ These authors and several others assume that TPPS_4_ is an achiral tecton^34^ thus puzzling results are presented throughout.

At low concentration and in initial stages after the pH drop, both ***M*** and ***P*** axial chiralities could occur at the sulfur atoms forming dimers or small oligomers. As aggregation proceeds beyond a critical nucleus stage, a homochiral propagation process appears to be thermodynamically favoured. This explains the apparent symmetry breaking of such TPPS_4_⋅2 HCl aggregates at later ripening stages. Under the critical size, detachment of TPPS_4_ molecules from heterochiral aggregates is probable with subsequent reattachment to the larger homochiral assemblies that autocatalyse their growth. In a very similar autocatalytic self‐assembly process, 14 BChl *c* monomers were found necessary to nucleate large tubular aggregates18a that were structurally well characterized by a combination of solid state NMR, cryo‐EM and molecular modeling.^35^

The obvious question is to ask where does, in a stagnant solution, the strong bias for a positive exciton couplet come from? We interpret the nonsilent ECD spectra just after injection into the aqueous HCl with pH 1 as being due to small chiral aggregates such as dimers, tetramers, and low oligomers which are present in the “as received” preparation. Most certainly, in the late production stages of these samples, solvents and water are removed on rotary evaporators. The rotating sense of these instruments can thus bias the chirality of these oligomeric species, strongly favouring one enantiomorphic form. When dissolving this material in pH 7 water, even though the monomeric species is predominant, small oligomers remain which will serve as nuclei in the dilution step associated with the pH drop, followed by ageing and ripening. It is after this nucleation phase that only one handedness will be preferred for the tube growth depending on the chirality of the pre‐existing nuclei.

To test this hypothesis, we employed the zwitterionic TPPS_4_ prepared as described earlier by ion exchange from the tetrasodium salt of TPPS_4_.^23^ The cation exchange resin replaces the sodium cations by H_3_O^+^. This lyophilized material, thus non‐rotary evaporated, dissolved in pH 7 distilled water when injected into pH 1 HCl, forms identical J‐aggregates as the TPPS_4_ ⋅ 2HCl described above. However, in this case, on a relatively smaller number of samples, we obtained a nearly 50:50 distribution of positive and negative exciton couplets for the 490 nm absorption maximum of the J aggregates. This points strongly towards the history of the sample preparation which can be the reason for biasing the chirality of the self‐assemblies. Melting experiments, as suggested by a kind referee, could help in annihilating pre‐existing nuclei.

Due to the relatively easy preparation of such tubes by our injection method using commercial samples, we foresee that for testing the resolution and setting up of new generation electron microscopes, TPPS_4_ aggregates will become a benchmark specimen.

In conclusion, the overall ordering in the 2D sheets very recently proposed by Ribó et al.^10^ is very similar to a collapsed version of our map at ∼5 Å resolution. Although their interpretation explains well the appearance of both J‐ and H‐aggregate bands, we demonstrate here that both chromatic shifts as well as their enigmatic chirality (see also Figure S4) are caused by TPPS_4_ aggregating at pH 1 as helical tubes with diameters averaging 160 Å where the individual porphyrins are arranged approximately normal to the axis of the tube. In our map, each monomer also appears to overlap its immediate neighbours, the sulphur atoms being stereogenic centers. Thus, we hope to have contributed to solving the long‐standing problem concerning the helical arrangement of TPPS_4_ molecules and their apparent symmetry breaking.

## Experimental Section

Experimental details are given in the Supporting Information.

## Supplementary Material

As a service to our authors and readers, this journal provides supporting information supplied by the authors. Such materials are peer reviewed and may be re‐organized for online delivery, but are not copy‐edited or typeset. Technical support issues arising from supporting information (other than missing files) should be addressed to the authors.

miscellaneous_information
