# Supplementary material for: Electron Cryo‐Microscopy of TPPS4⋅2HCl Tubes Reveals a Helical Organisation Explaining the Origin of their Chirality
Source: Chemphyschem. 2013 Jul 31;14(14):3209–14. doi: 10.1002/cphc.201300606 (PMC4281918; doi:10.1002/cphc.201300606)
Supplement: Supplementary file 1 — miscellaneous_information [file cphc-14-3209-s6.pdf]

CHEMPHYSCHEM

Supporting Information

© Copyright Wiley-VCH Verlag GmbH & Co. KGaA, 69451 Weinheim, 2013

Electron Cryo-Microscopy of $TPPS_4 \cdot 2HCl$ Tubes Reveals a Helical Organisation Explaining the Origin of their Chirality**

Judith M. Short,*[a] John A. Berriman,[b] Christian Kübel,[b] Zoubir El-Hachemi,[c]
Jean-Valère Naubron,[d] and Teodor Silviu Balaban*[e]

cphc_201300606_sm_miscellaneous_information.pdf

Supporting Information

Electron cryo-microscopy of *meso*-tetrakis-(4-sulphonatophenyl)-porphyrin dihydrochloride tubes reveals a helical organisation that explains the origin of their chirality

Judith M. Short,*,[a] John A. Berriman,[b] Christian Kübel,[b] Zoubir El-Hachemi,[c] Jean-Valère Naubron,[d] and Teodor Silviu Balaban*,[e]

[a] Division of Structural Studies, MRC Laboratory of Molecular Biology, Francis Crick Avenue, Cambridge Biomedical Campus, Cambridge, CB1 0QH, United Kingdom, E-mail: jms@mrc-lmb.cam.ac.uk

[b] Karlsruhe Institute of Technology (KIT), Institute of Nanotechnology (INT), Hermann-von-Helmholtz-Platz 1, Building 640, D-76344 Eggenstein-Leopoldshafen, Germany

[c] Department of Organic Chemistry, University of Barcelona, Catalonia, Spain

[d] Aix Marseille Université, CNRS FR 1739, Spectropole, Avenue Escadrille Normandie Niemen, Marseille, France

[e] Aix Marseille Université, Institut des Sciences Moléculaires de Marseille (*iSm2*), CNRS UMR 7313, Chirosciences, Avenue Escadrille Normandie Niemen, CaseA62, F-13397 Marseille CEDEX 20, France. E-mail: ts.balaban@univ-amu.fr

Table of Contents

Materials and Methods............8
Preparation of 5,10,15,20-Tetrakis(4-sulfonatophenyl)porphyrin free of the meta-sulfonated isomer ..8
HPLC Analysis............9
Electron Microscopy............9
Image Processing............9
Electron Diffraction............9
X-ray Diffraction............10

Figure S1. Additional UV-Vis absorption spectra............11

Figure S2. Measurements of tube radii............12

Figure S3. Indexing the diffraction pattern............13

Figure S4. Image of the map showing the directions of the J and H aggregates transition dipole moments..14

Materials and Methods

$TPPS_4$ • 2HCl was purchased from Frontiers Scientific Inc., Logan, Utah, while $TPPS_4$ hydrate was obtained from TCI-Europe, Belgium. These materials were used without any further purification although the reverse phase HPLC traces of the former material showed variable amounts of a slower eluting impurity in an amount which varied from batch to batch. Zwitterionic $TPPS_4$ was prepared as described earlier[23] and a modified procedure is detailed below. Distilled or nano-electronics quality water from a Millipore system was used to prepare stock solutions of $TPPS_4$ which were slowly injected, preferably through a glass capillary, into a much larger volume (> 100) of pH = 1 HCl. The concentrated acid solutions of HCl (Merck 37 %) were employed as received to prepare the pH = 1 solutions presuming these were free of any chiral contaminants. For obtaining tubes and intense ECD signals the solutions were aged for at least 24 h in the dark. UV-Vis-NIR Spectra were measured at KIT on a Varian Cary 500 spectrophotometer and in Marseille on a Shimadzu UV-2401 (PC) instrument. ECD spectra were measured in Marseille on a JASCO 815 spectrometer in quartz cuvettes of different pathlengths allowing the strongly absorption solutions to pass enough light to the detector so that meaningful and reproducible spectra could be obtained. The samples were thermostated at 20.0 ± 0.2 °C by means of a PTC 423 Peltier holder.

Preparation of 5,10,15,20-Tetrakis(4-sulfonatophenyl)porphyrin free of the *meta*-sulfonated isomer[23] obtained from 5,10,15,20-Tetrakis(4'trimethylsilylphenyl)porphyrin $TPP(SiMe_3)_4$ by treatment with the sulfonation reagent, $ClSO_3SiMe_3$.

$TPP(SiMe_3)_4$ and $TPPS_4$.

The synthesis was based on the method of Ye and Naruta.[1] To a 2 L round-bottomed flask containing CH_2Cl_2 (1 L) purged with N_2 (15 min) were added by syringe 2,2-dimethoxypropane (1 mL), pyrrole (1.01 mL), 4-trimethylsilylbenzaldehyde (2,7 g) and BF_3•OEt_2 (0.19 µL). The mixture was stirred at room temperature under Ar atmosphere for 1 h. 2,3-Dichloro-5,6-dicyano-1,4-benzoquinine (DDQ, 2.5 g) was then added and the solution was further stirred at room temperature and exposed to air for 1 h. Most of solvent was then eliminated by rotary evaporation and the mixture was filtered through Florisil (17 x 6 cm) and eluted with CH_2Cl_2. The residue of this filtrate was subjected to chromatography on a silica-gel column (15 x 6 cm). $TPP(SiMe_3)_4$ was eluted with CH_2Cl_2 : Hexane (1:1), rotary evaporated and dried upon paraffin.

Powdered $TPP(SiMe_3)_4$ (130 mg) was dissolved in anhydrous CCl_4 (30 mL) in a 100 mL round-bottomed flask under Ar atmosphere. Then 0.3 ml of $ClSO_3SiMe_3$ was added and the resulting mixture was heated to reflux overnight. After cooling, the reaction was quenched by the addition of NaOH 1 N (15 mL) and was stirred at room temperature for further 30 min. The residue obtained by rotary evaporation was purified by repetitive column chromatography in reverse phase (SiO_2-C18) using a water/methanol gradient (2:1) and finally lyophilized.

[1] B.-H. Ye, Y. Naruta, *Tetrahedron*, **2003**, *59*, 3593-3601.

HPLC analysis

The purity of the porphyrin fractions was tested by HPLC on a Nucleosil 120-5C18 column, using a gradient from methanol/tetrabutylammonium phosphate buffer (3 mM, pH 7, 1/1, v/v) to pure methanol at a flow rate 0.6 mL/min (41 min, t_R= 16.4 min). The slower eluting impurity (t_R = 16.9 min) which is present in commercial samples was absent in this preparation. This impurity is presumably a $TPPS_4$ isomer having one phenyl group sulphonated in the *meta*-position.

Electron microscopy

A 10 mM stock solution was made in double distilled water of the $TPPS_4$ 2HCl solid (Frontiers Scientific, Ohio). A 50 µL aliquot of this was slowly pipetted into 5 mL of 0.1 N HCl stirred vigorously in a test tube on a vortex mixer. After storage of this solution for several days, 5 µL drops were pipetted onto Quantifoil (Jena, Germany) gold R0.6/1 holey grids which had been glow-discharged before use to improve surface hydrophilicity. The grids were blotted by hand and plunge frozen into liquid ethane on a laboratory-built freezing device. Following storage under liquid nitrogen, grids were later transferred into the FEI (Eindhoven, The Netherlands) G2 Polara TEM at the Laboratory of Molecular Biology (Cambridge, UK) operating at liquid nitrogen temperature. The accelerating voltage was 300keV and the nominal magnification was 114,000 fold. Micrographs were taken under low-dose conditions with an electron dose approximating 40 e/$Å^{-2}$ and an underfocus range of 1.9 – 4.2 µm. The images were recorded on Kodak SO-163 film and developed in full strength Kodak D-19 developer for 12 min at 21°C.

Image processing

The electron micrographs were digitized by a home built KZA scanning densitometer[2] using a step size of 6µ, which corresponds to 0.53Å on the specimen. Tubes were selected from the digitized images based on the quality of their power spectra. Using Ximdisp,[3] each tube was boxed, padded to 4096 x 4096 pixels and floated. They were then vertically aligned and compressed via local averaging by a factor of 2 to a working pixel resolution of 1.052Å. Tube images were corrected for contrast transfer function and their Fourier transforms calculated (Figure 3). Indexing the lattice was aided by correcting for out-of-plane tilt angles and horizontal shift calculations using the helical programs from the MRC image processing software.[4] Layer line data from four layer lines were extracted from both sides of each tube, corrected for in-plane rotation and shear. They were then fitted together and averaged before inverse Fourier transformation to produce a three-dimensional (3D) structure

Electron diffraction

The same specimen preparation method was used as for electron microscopy, except that following ageing of the $TPPS_4$ suspension in HCl, a 1.5 mL aliquot was transferred to a tube used in a TLA-55 rotor of a Beckman (Beckman Coulter, USA) Optima TL series bench-top Ultracentrifuge. The sample was then spun at 55,000 rpm for two hours and the pellet was resuspended in 100 µL of the supernatant by gently passing it in and out of the tip of a

micropippete. Following this flow alignment method, 5 µL of the concentrated sample was passed onto the grid's surface taking care to maintain any alignment and the grids were frozen as described before. These grids were later examined on a FEI (Eindhoven, The Netherlands) Cs-corrected Titan at the Institute of Nanotechnology (KIT, Karslruhe, Germany) at 300 keV using a Gatan (Pleasanton California USA) 914 double tilt coldstage. Low dose diffraction methods included a Selected Area aperture to limit the region contributing to the pattern to those showing best alignment in the ice. Camera lengths were chosen between 0.6 to 1.5 meters and recorded on a Gatan UltraScan 1000 CCD camera. The recorded spacings were calibrated against a thallous chloride calibration grid exposed under identical electron-optical conditions.

X-ray Diffraction

In order to obtain a sufficiently concentrated sample, the same centrifugation method was used as for electron diffraction except that 24 pellets were pooled into 1.5 mL and this concentrated sample was again centrifuged at 55,000 rpm for two hours. The final pellet was resuspended in 0.5 mL some of which was flow-aligned through a fine capillary into MiTeGen (Ithaca, NY, USA) Micro-RT capillary mount RT-T1 plastic "straw" sealed with wax. X–ray reflections were recorded on a Rigaku (Tokyo, Japan) MicroMax-007 HF diffractometer using an R-Axis IV detector. Camera lengths of 150 and 300 mm were used and exposure times of 1 to 15 minutes. Spacings were calibrated against a LaB_6 standard mounted as a powder in a MiTeGen straw.

[2] R. Henderson, D. Cattermole, G. McMullan, S. Scotcher, M. Fordham, W. B. Amos, A. R. Faruqi, *Ultramicroscopy,* **2007**, *107*, 73-80.

[3] J. M. Smith, *J. Struct. Biol.* **1999**, *125*, 223-228.

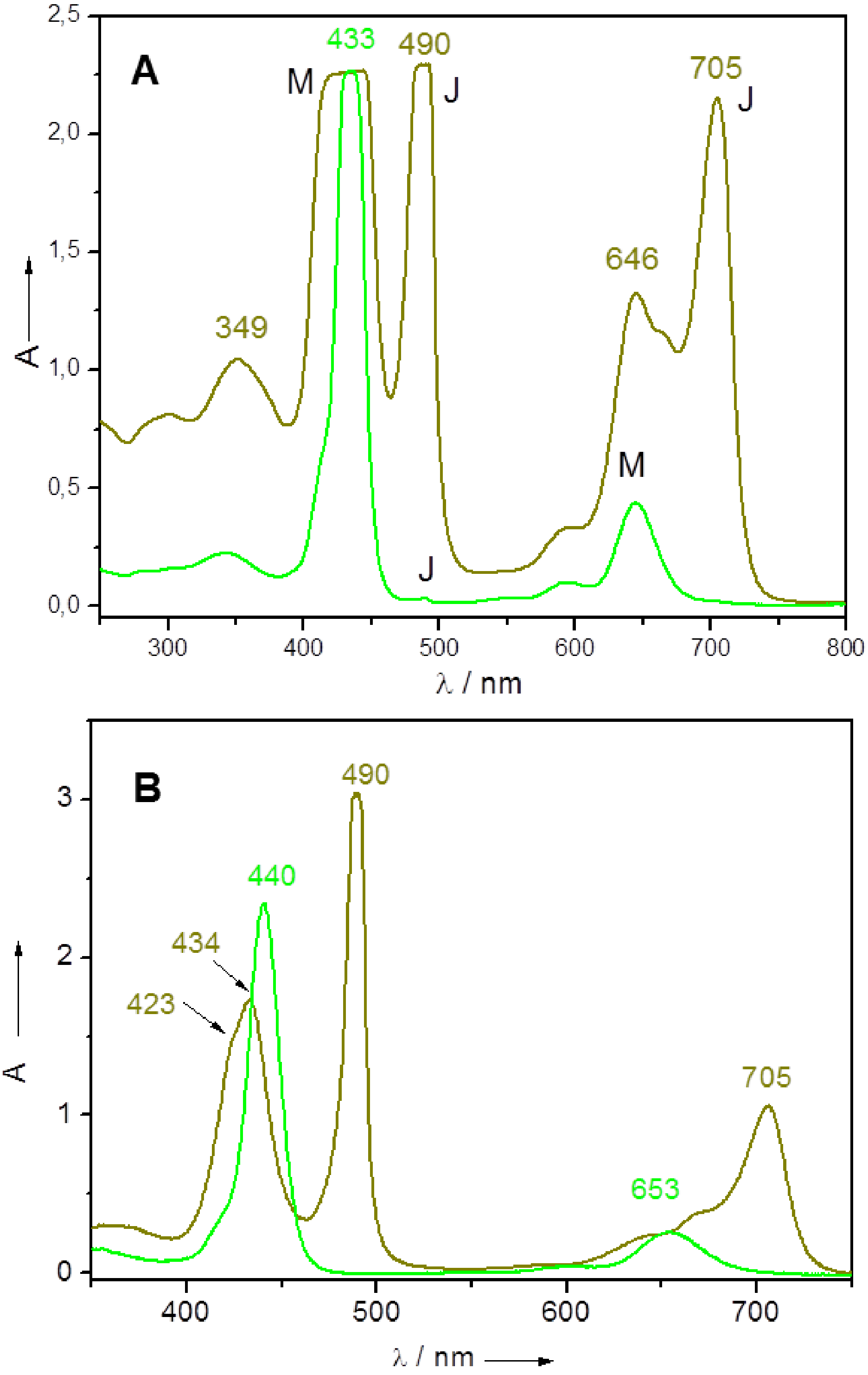

Figure S1. **Additional UV-Vis Absorption spectra**. **A.** Gold trace: Dilution of the same solution presented in the Main Text Figure 1A by an equal volume of 100 mM HCl. Note the increase of the monomer maxima at 646 and 433 nm at the expense of the J aggregate maxima at 705 and 490 nm. The 433 and 490 maxima are truncated. Green trace. Same solution after dilution with a five-fold volume of 100 mM HCl. **B**. Gold trace: Typical aggregate spectrum formed by injecting a concentrated $TPPS_4$ solution in pH = 7 distilled water into a much larger volume of 100 mM HCl. Note that the monomer maximum at 646 nm is much diminished in comparison with the golden trace above in panel A. Green trace : same amount of $TPPS_4$ dissolved in pH = 7 distilled water injected this time into ~10 M HCl. The 440 and 653 nm maxima are due to a monomeric form with all four SO_3H groups protonated. Pathlength was 5 mm.

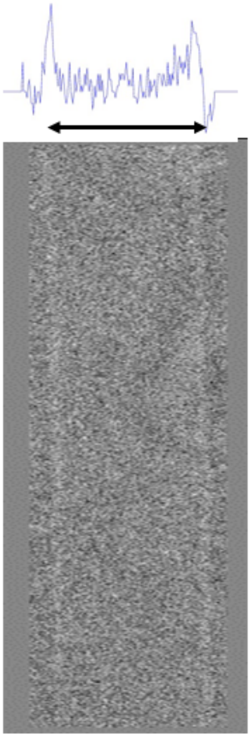

Figure S2. **Measurements of tube radii**. A single tube and its summed density trace demonstrates the tube profile. The black arrowed line indicates the maximum tube width of 160 Å.

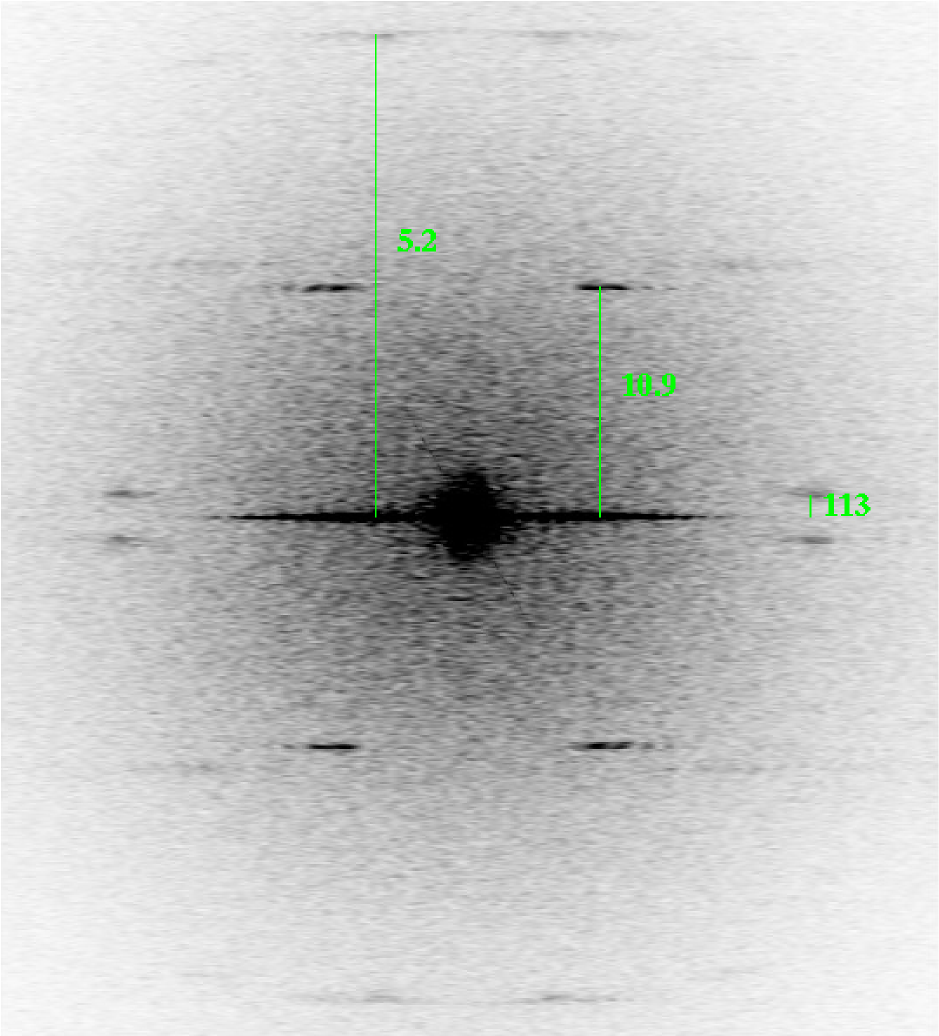

Figure S3. **Indexing the diffraction pattern**. To improve the signal-to-noise ratio and make indexing the diffraction pattern as accurate as possible, the power spectra of 116 tubes were summed. The layer line reflections are clearly defined, which indicates reasonably homogeneous symmetry. Bessel orders and the repeat distance for the reconstruction were estimated from this average. The layer line spacings are shown in reciprocal Ångströms.

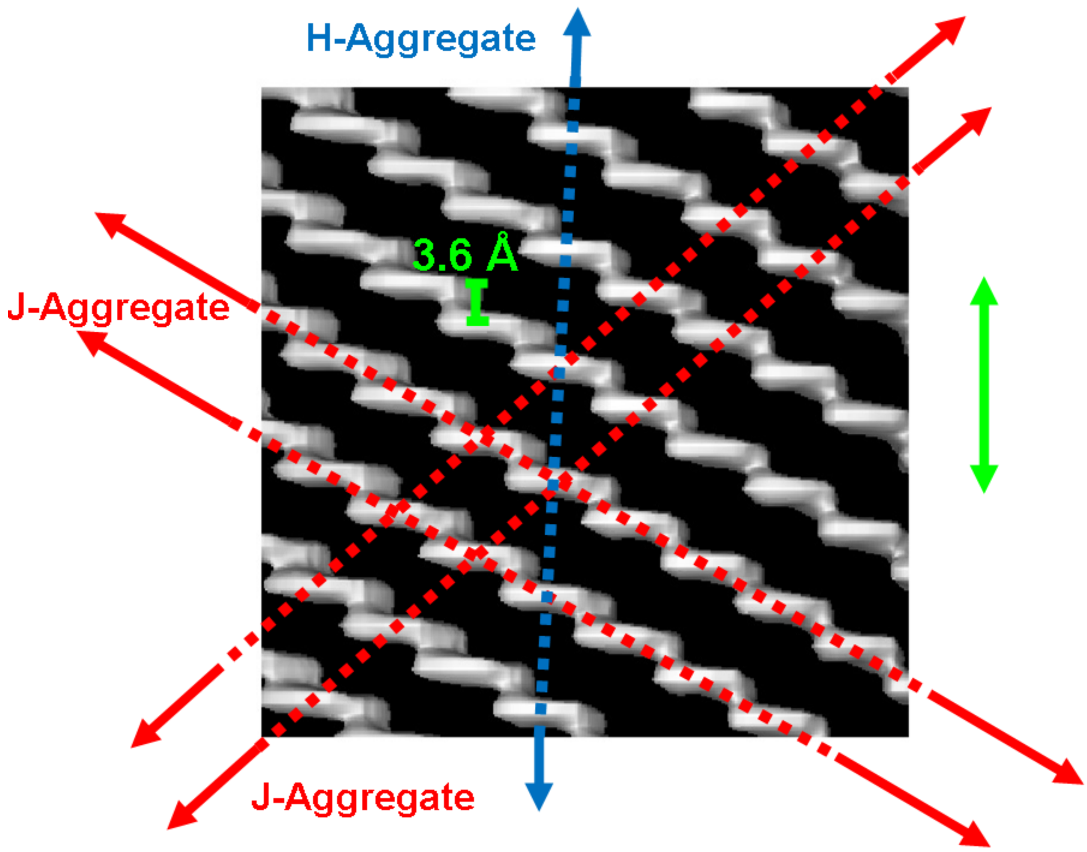

Figure S4. Image of the map showing the directions of the J and H aggregates transition dipole moments. Schematic illustration, extracted from a surface calculation of the 3D-map, of the J- (red-shifted) versus the H-aggregate (blue-shifted) couplings between neighbouring $TPPS_4$ molecules. The direction of the tube axis is shown by the vertical green double headed arrow. The green scale bar was calculated directly from the 3D-map of an ***M*** (–) 26-start helix. The interested reader is referred to the very similar Figure 5 in the recent publication listed as ref. 10 in the main text [Z. El-Hachemi, C. Escudero, F. Acosta-Reyes, M. T. Casas, S. Aloni, V. Altoe, G. Oncins, A. Sorrenti, J. Crusats, J. L. Campos, J. M. Ribó, *J. Mater. Chem. C*, **2013**, *1*, 3337-3346. DOI: 10.1039/C3TC30299G.].
